# A rare presentation of carcinosarcoma of the bone in a young female; response with gemcitabine and docetaxel

**DOI:** 10.1186/s13569-019-0120-7

**Published:** 2019-07-02

**Authors:** Nicholas King, Keshav Kukreja, Albina Murzabdillaeva, Yasir Ali, Jason Willis, Abhishek Maiti, Hilary Ma, Joan Bull

**Affiliations:** 1 University of Texas Health Sciences Center at Houston, McGovern Medical School, Houston, TX USA; 2 University of Texas Health Sciences Center MD Anderson Cancer Center, Houston, TX USA

**Keywords:** Carcinosarcoma, Sarcomatoid carcinoma, Bone, Gemcitabine, Docetaxel

## Abstract

**Background:**

Sarcomatoid carcinoma, or carcinosarcoma, is a neoplasm that contains both sarcomatous and carcinomatous elements. It is an extremely rare cancer most often arising from visceral organs. Here we report the seventh documented de novo case of carcinosarcoma of the bone, in a young female who showed initial clinical improvement with gemcitabine and docetaxel.

**Case presentation:**

A 36-year-old Caucasian female presented with diffuse musculoskeletal pain that had progressed from her shoulder to her back, arm, and knee over 6 months. Imaging revealed diffuse sclerotic lesions of bilateral humeral heads, iliac and ischial bones, and thoracic and lumbar spine. Histopathologic examination of biopsies from the T9 vertebra and left femur showed mainly sarcomatous spindle cells with focal osteoid production. Immunostaining showed the cells to be OSCAR cytokeratin, patchy positive for pankeratin, and negative for CK7, GATA3, S100, SOX10, CD99, EMA, AE1/AE3, and HMW keratin indicative of an epithelial origin. After thorough clinical correlation, sarcomatoid carcinoma of a visceral organ was excluded and the diagnosis of primary sarcomatoid carcinoma of the bone was ultimately favored. She received chemotherapy with gemcitabine and docetaxel, and showed improvement at 6 months but ultimately passed 1 year post diagnosis.

**Conclusions:**

Primary carcinosarcoma of the bone is an extremely rare malignancy. Early diagnosis is crucial as localized disease may be curable with resection. As shown in this case, combination chemotherapy with gemcitabine and docetaxel is a potential option in patients with unresectable or metastatic disease.

## Background

Sarcomatoid carcinoma, also known as carcinosarcoma, is a high-grade malignant neoplasm that contains both carcinomatous and sarcomatous components [1, 2]. It generally occurs in the gastrointestinal or genitourinary system, with occasional metastasis [3–5]. Primary de novo bone sarcomatoid carcinoma of the bone is exceedingly rare, with only six reported cases in the literature [6–11]. Here we report the seventh documented case of sarcomatoid carcinoma of the bone, with diffuse metastasis throughout the bony skeleton.

## Case presentation

A 36-year-old Caucasian woman with no past medical history presented to the emergency department with progressive, diffuse musculoskeletal pain that was dull in character. Initially the pain was localized to the right shoulder, but over 6 months progressed to her back, arm, and knee.

**Fig. 1:**
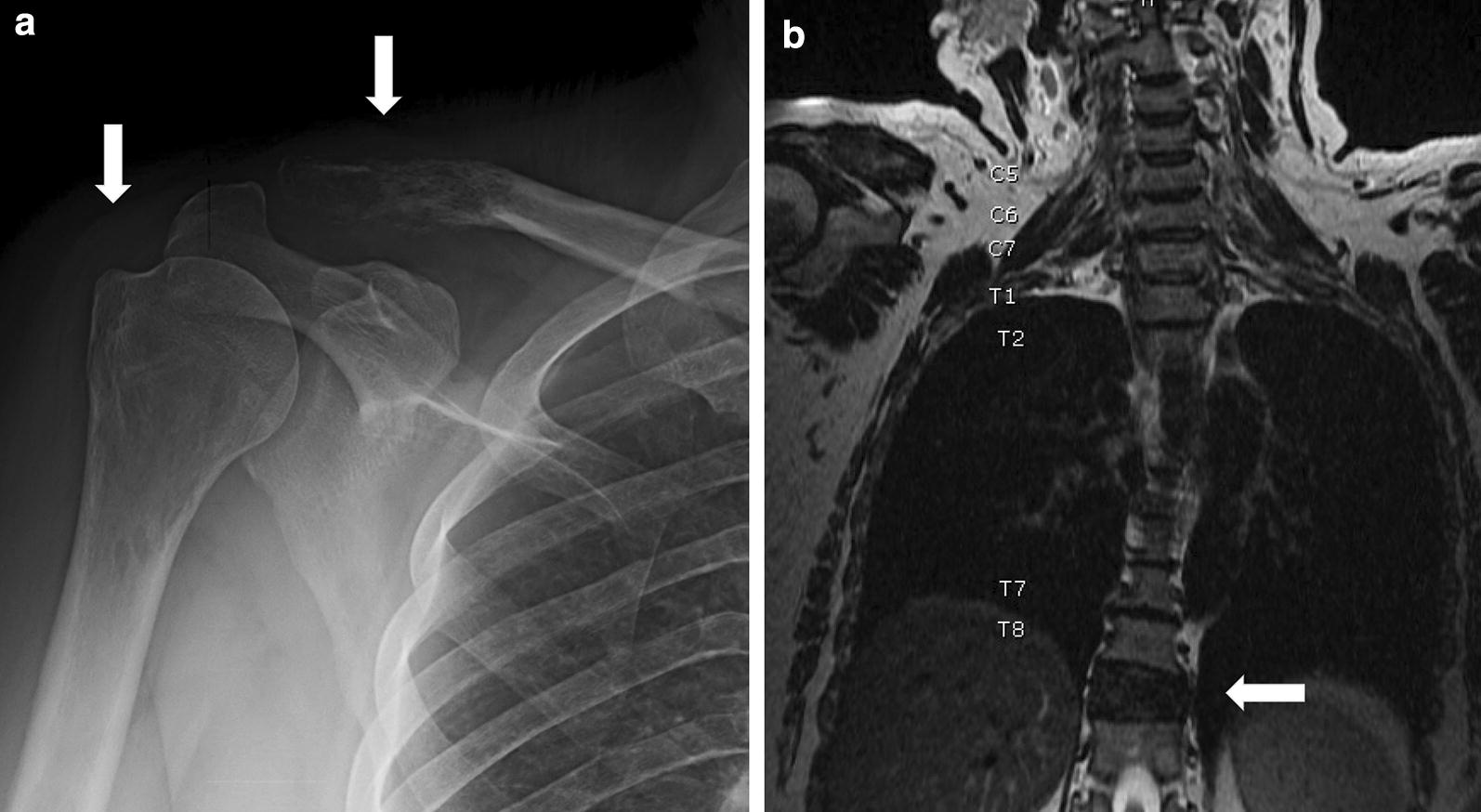
Initial radiography of Lesions. **a** Plain film radiography showing the lytic lesions present in the right distal clavicle and humeral head (arrows). **b** T1 MRI of the chest showing the T9 pathologic fracture (arrow)

Radiographs showed multiple lytic lesions (Fig. 1a), and subsequent computed tomograms (CT) revealed extensive lytic lesions to bilateral humeral heads, iliac bones, ischial bones, thoracic spine and lumbar spine with pathologic fracture of T9. Due to new-onset numbness and tingling of her leg, emergent magnetic resonance image (MRI) was obtained which confirmed extensive metastases to the left femur with distal non-displaced diaphysis pathologic fracture, in addition to metastases to the humerus, scapula, clavicle, 4th and 5th ribs, throughout the pelvis, and the spine with pathologic T9 fracture and mild spinal canal stenosis but no cord compression (Fig. 1b). She underwent left femur fixation by retrograde intramedullary nailing. Whole body positron emission tomography CT (PET/CT) showed extensive hypermetabolic metastasis to the bony skeleton (Fig. 4a), however a non-osseous primary was never identified despite thorough clinical and radiologic evaluation.

**Fig. 2:**
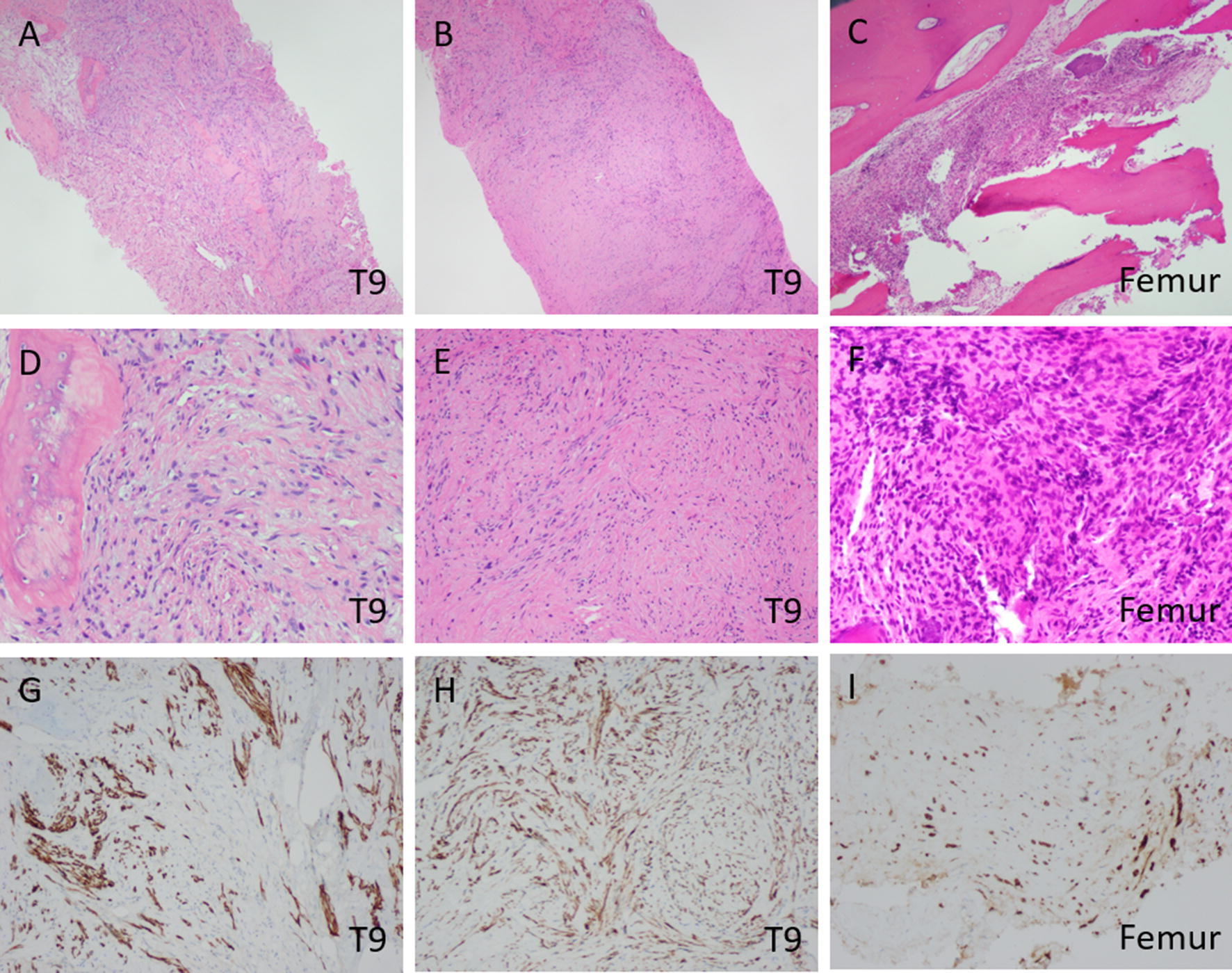
Staining of Specimens. Histopathologic features of the T9 biopsy and intraoperative femur stabilization specimens, indicated in lower right corners. **A** Nests of osteoid producing cells can be seen surrounded by swirls of pleomorphic spindle cells and reticular substance. HE ×40. **B** The tumor is predominantly made up of irregular spindle cells. HE ×40. **C** The tumor can be seen infiltrating normal bone architecture, HE ×40. **D** Pleomorphic spindle cells with intervening stroma. HE ×200. **E** Poorly defended clusters of cells can be seen surrounded by neoplastic stroma, HE ×200. **F** Spindle cells showing a high degree of pleomorphism, hyperchromatic nuclei, and irregular nuclear contours. HE ×200. **G** Positive immunohistochemical staining for OSCAR cytokeratin in scattered spindle cells, ×200. **H** Scattered positive immunohistochemical staining for OSCAR cytokeratin, ×200. **I** The same scattered positive immunohistochemical staining for OSCAR cytokeratin is seen in the femur biopsy, ×200

**Fig. 3:**
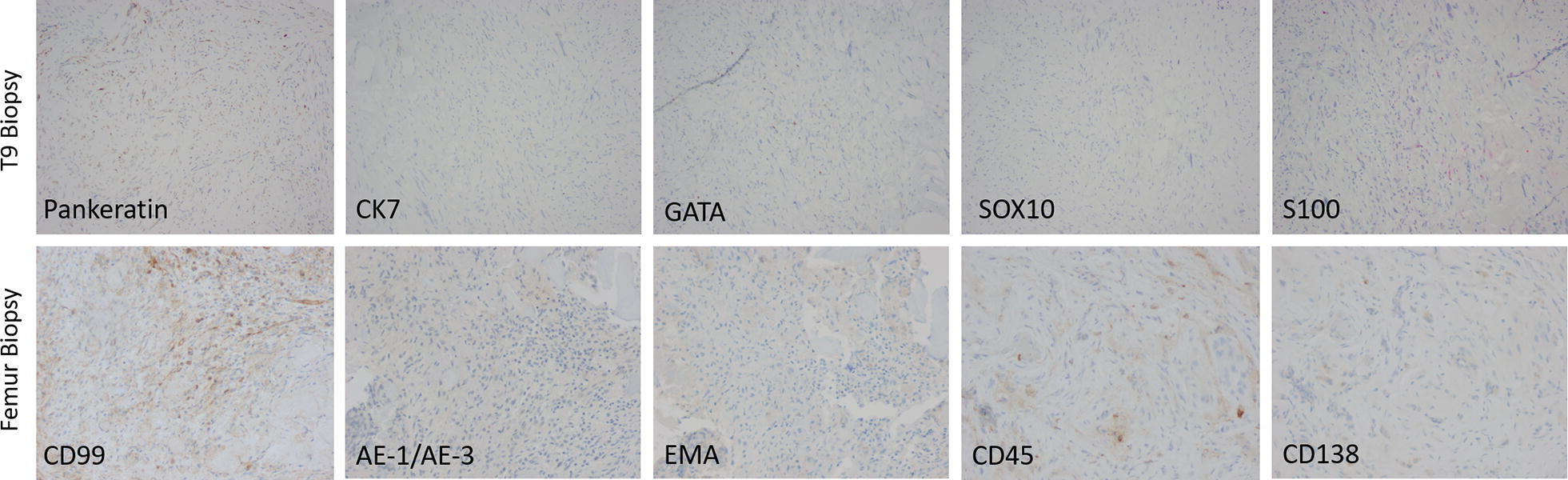
Additional immunostaining of specimens. Immunohistochemical features of the T9 biopsy and intraoperative femur stabilization specimens indicated at left, all ×200. Pankeratin is weakly positive in a subset of spindle cells; CK7, GATA, SOX10, and S100 all showed no reactivity in the T9 biopsy specimen. Additional stains performed on the specimen from the femur stabilization are non-reactive for CD99, AE-1/AE-3, EMA, CD45, and CD138

Histopathologic examination of a biopsy from the T9 lesion revealed proliferation of spindle cells with hyperchromatic, pleomorphic nuclei, and irregular nuclear contours organized in swirls giving a nested appearance (Fig. 2A, B, D, E). Occasional mitoses but no confluent necrosis can be seen. Focal areas of osteoid production and large amounts of reticular substance production can be seen (Fig. 2A, D). This morphology and activity of the spindle cells was suggestive of a mesenchymal origin of the tumor. However, immunohistochemical staining showed the atypical spindle cells to have scattered staining for OSCAR cytokeratin, with weakly and patchy positivity for pankeratin, hallmarks of an epithelial origin (Fig. 2G, H). CK7 was negative ruling out synovial sarcoma, metastatic carcinoma of the thyroid, breast, gastrointestinal, pancreatic, renal, bladder, urothelial, cervical, and ovarian origins. GATA3 was negative further ruling out metastatic carcinoma of the breast and urothelium; and excluding sarcomatoid mesothelioma, choriocarcinoma, and gestational trophoblastic tumors. SOX10 did not show any staining excluding soft tissue tumors of neural crest origin and melanoma. S100 staining was absent eliminating the possibilities of melanoma, dendritic cell tumors, myoepithelial tumors, neural tumors, chondroid tumors (Fig. 3, Table 1). These findings were suggestive of a malignant sarcomatoid neoplasm, which could represent the process of metastasis to the bones due to patient’s clinical presentation of multifocal lytic bone lesions. But after thorough clinical correlation and review of all available imaging studies, sarcomatoid carcinoma of a visceral organ was excluded and a final diagnosis of primary sarcomatoid carcinoma of the bone was favored. Histopathologic examination of the intraoperative femur biopsy showed a similar cytomorphologic profile to the T9 biopsy (Fig. 2C, F, I). Additional stains performed on this section showed the neoplastic cells to be positive for OSCAR (Fig. 2I) and negative for CD99 and EMA, further ruling out epithelioid sarcoma, synovial sarcoma, and mesenchymal chondrosarcoma. Staining for AE1/AE3, CD45, and CD138 were negative (Fig. 3, Table 1). These findings were also congruent with the diagnosis of sarcomatoid carcinoma.

**Table 1:** Immunostaining summary

OSCAR cytokeratin	Scattered positivity in both specimens
T9 biopsy	
Pankeratin	Weakly and patchy positive
CK7	Negative
GATA	Negative
SOX10	Negative
S100	Negative
Femur biopsy	
CD99	Negative
AE-1/AE-3	Negative
EMA	Negative
CD45	Negative
CD138	Negative

Immunohistochemical stains and outcomes of the T9 and femur biopsies. The stains were consistent with a neoplasm of epithelial origin

**Fig. 4:**
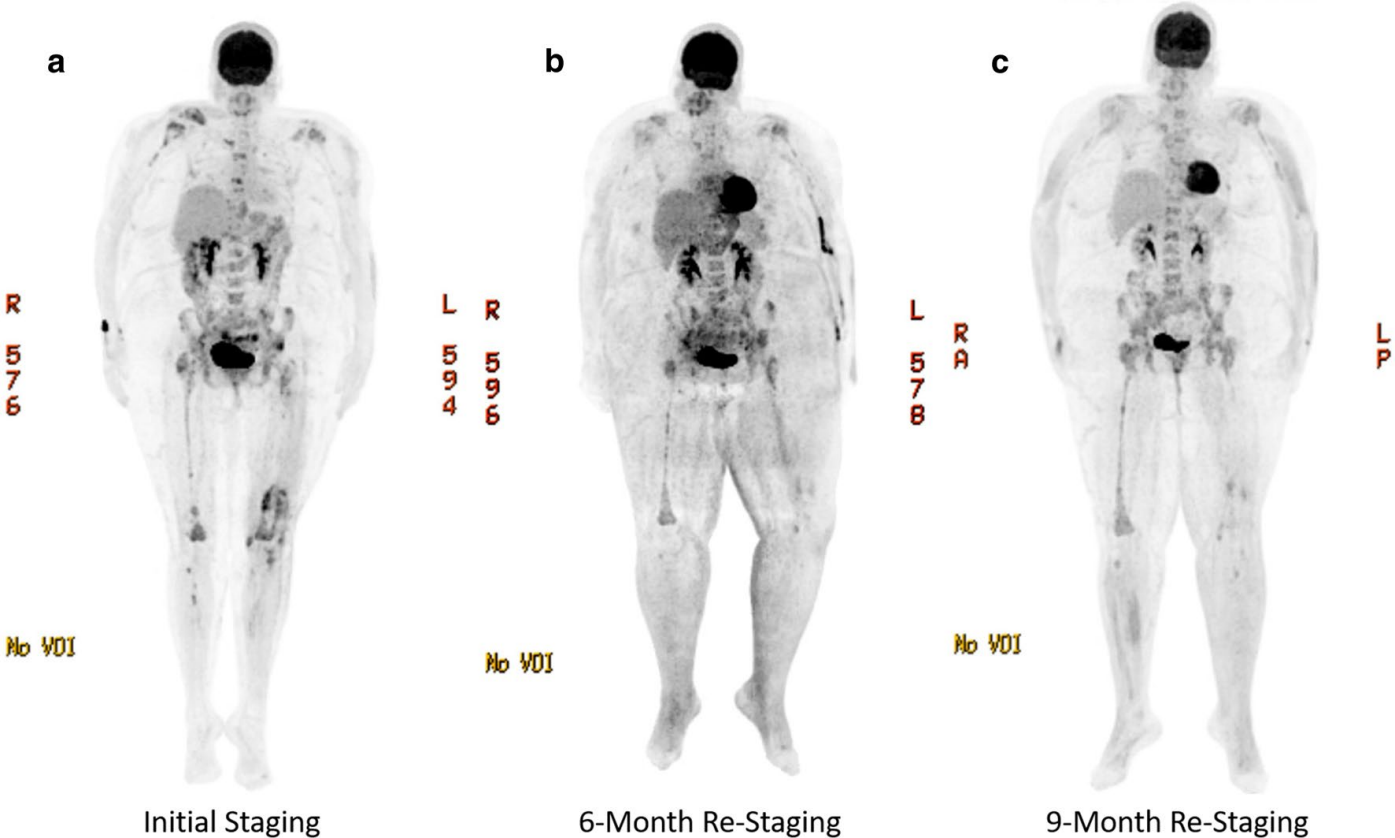
Radiographic PET/CT staging of lesions. The delayed enhancement PET scan shows 18-fluorodeoxyglucose (FDG) uptake over time. Metabolically active organs such as the bladder, kidneys, testicles and brain show increased uptake normally. **a** Initial staging PET/CT showing lesions in the bilateral humeri, claviculi, distal femurs, proximal tibias, pelvis, and spine. **b** 6-month re-staging PET/CT showing regression of lesions in bilateral femurs, right shoulder, and multiple levels of the thoracic spine consistent with a chemotherapeutic response. **c** 9-month re-staging PET/CT showing multifocal progression of osseous lesions

Due to the multifocal nature of the disease, the patient was not a candidate for curative resection. External beam radiotherapy was employed to prevent worsening of the spinal stenosis. Systemic chemotherapy with eight cycles of gemcitabine 600 mg/m^2^ and docetaxel 25 mg/m^2^ every 2 weeks was planned, however the patient only received infusions every 4 weeks due to delays. At 6-month follow-up, she had completed four cycles of chemotherapy and showed a positive treatment response with decreased hypermetabolism of bony lesions (Fig. 4a, b). However, at 9 months follow up following six cycles, PET/CT showed progression of the lesions, with multifocal areas of increased metabolic activity throughout the spine, sacrum, and pelvis (Fig. 4b, c). Salvage therapy was attempted with paclitaxel 175 mg/m^2^ and carboplatin 250 mL every 21 days however the patient only completed one cycle before passing away 1 year after her diagnosis. An autopsy was not performed.

## Discussion and conclusions

The description of carcinosarcoma, or sarcomatoid carcinoma, first laid out by Virchow, is that of a neoplasm containing both carcinomatous and sarcomatous components [1]. Carcinosarcoma could be the product of two separate primary tumors (the polyclonal theory), a carcinoma and a sarcoma, arising as the same time and subsequently growing together [12, 13]. Alternatively, it could arise from a single progenitor cancer cell (monoclonal theory) that undergoes an epithelial to mesenchymal (EMT) or mesenchymal to epithelial (MET) transition to give rise to distinct lineages [13, 14]. While reports of two distinct primaries occur [13], a growing body of evidence has shown that a monoclonal, EMT/MET-derived multilineage neoplasm with both sarcomatous and carcinomatous components is the more common cause of carcinosarcomas [15–17].

Carcinosarcoma is a rare neoplasm that has been reported in many organs including the gastrointestinal system, genitourinary system, and pulmonary tree [3–5, 18]. The ratio of epithelial to sarcomatous components varies, as does the relative degree of differentiation of the cells [19]. The general prognosis for carcinosarcomas is poor due to its high grade [20, 21].

Primary carcinosarcoma of the bone is extremely rare and has only been described a handful of times in the literature. Hutter et al. [22] likely encountered it in their case series on mixed-lineage sarcomas of bone in 1966, identifying 4 tumors with sarcomatous and carcinomatous elements. Most cases to date (Table 2) have described focal lesions occurring at the ends of long bones (the humerus, femur, tibia, and fibula) composed of co-mingled sarcomatous and epithelioid components [6–9, 11]. All were treated with resection of the tumor focus, except the cases described by Frydman et al. and Shiraishi et al. which received delayed resection and metastasized to the lungs [7, 9]. The cases of focal lesions treated with resection demonstrated durable remission without evidence of recurrence [6, 8, 11] suggesting an indolent nature of this entity with low propensity for early microscopic metastases. This indicates that for focal carcinosarcoma of the bone, primary resection of the early lesion can potentially cure the patient. Paczos et al. describe a carcinosarcoma of the bone that presented with diffuse skeletal lesions composed of undifferentiated spindle cells almost exclusively, and after receiving 5 rounds of MAID chemotherapy and radiation therapy to the spine continued to deteriorate at 1 year [10, 23].

**Table 2:** Carcinosarcoma of bone cases summary

Case	Age/gender	Primary site (s)	Histopathology	Metastasis	Treatment	Outcome
Focal carcinosarcoma of the bone						
Ling and Steiner 1986 [6]	68-female	Humerus	Chondrosarcoma and SCC	None	Resection	Disease free at 3 years
Frydman et al. 1991 [7]	42-male	Tibia	Lymphoma-like round cells, osteogenic sarcoma, and spindle cells	Lungs	Chemotherapy appropriate for a non-Hodgkin’s lymphoma	Succumbed to lung metastases over a year post-diagnosis
Kramer et al. 1993 [8]	13-male	Tibia	Osteogenic sarcoma and epithelial differentiation	None	Resection, neoadjuvant methotrexate, doxorubicin, and cisplatin	Disease free at 1 year
Shiraishi et al. 2005 [9]	53-male	Femur	Chondrosarcoma and SCC	Lung and soft tissue	Curettage, delayed resection, paraplatine 450 mg once a month for 6 months	Succumbed to pulmonary metastases 6 months post-diagnosis
Ishida et al. 2014 [11]	59-female	Fibula	Chondrosarcoma, SCC, and spindle cells	None	Resection	Disease free at 1 month
Diffuse carcinosarcoma of the bone						
Paczos et al. 2009 [10]	63-male	T10-L1 vertebrae, left iliac body	Poorly differentiated spindle cells	Multiple bony lesions	Radiotherapy, 5 courses of MAID protocol chemotherapy	Disease progression at 1 year
Present case	36-female	Bilateral humeri, pelvis, thoracic lumbar spine	Nondescript spindle cells	Extensive bony infiltration	Gemcitabine 600 mg/m^2^ and docetaxel 25 mg/m^2^, followed by salvage chemotherapy	Reduced tumor burden at 6 months, expired at 1 year post-diagnosis

*SCC* squamous cell carcinoma

In this report, we report the 7th documented case of sarcomatoid carcinoma, or carcinosarcoma, arising from bone. This case presented with diffuse pain that was revealed to be multifocal skeletal lesions. The neoplasm was very poorly differentiated, with predominantly sarcomatous spindle cells similar to the cases described by Paczos et al. [10]. This patient underwent palliative radiotherapy and chemotherapy with gemcitabine and docetaxel and showed a therapeutic response at 6 months, but ultimately passed 1 year after diagnosis [24].

Given the rarity of primary carcinosarcoma of the bone, the natural history of this disease is unknown. The cases reported in literature suggest that primary carcinosarcoma of the bone may be a heterogenous entity showing two distinct phenotypes. One group of patients present with focal lesions within long bones and are composed predominantly of sarcomatous and epithelioid elements [6–9, 11]. The other group presents with diffuse lesions, such as the one described here and by Paczos et al. [10]. The diffuse lesions are composed predominantly of poorly differentiated spindle cells, and diffusely involve the skeleton. The focal carcinosarcoma of the bone appears to stay localized to its site of origin initially, allowing for curative resection. Three cases of local carcinosarcoma of the bone were cured by local resection [6, 8, 11], while the others succumbed to metastases to the lungs [7, 9]. The delay in treatment for the cases reported by Frydman et al. and Shiraishi et al. hint that the risk of metastasis increases with time [7, 9].

Carcinosarcoma of the gastrointestinal and genitourinary systems carry a poor prognosis [20, 21], however the prognosis of carcinosarcoma of the bone appears to be stratified by its characterization as metastatic vs. local. Focal carcinosarcoma of the bone appears to be curable by early detection and resection [6, 8, 11], however, as with other cancers, becomes uncurable once metastasis occurs [7, 9]. For patients with localized disease, most (3/5) were disease free at follow up, while the others (2/5) succumbed to lung metastasis by 1 year. The cases that presented with diffuse carcinosarcoma of the bone (Paczos et al. [10] and this report) all required multiple rounds of chemotherapy and radiation, and were unable to induce full remission. Our patient was treated with gemcitabine and docetaxel which showed initial activity against the neoplasm, and might represent an efficacious therapeutic approach with optimal dose density [24].

This report therefore presents the documented 7th de novo case of bone-derived sarcomatoid carcinoma, and the 2rd documented case to present with diffuse bony lesions. This patient’s disease followed a course similar to the other case of diffuse carcinosarcoma of the bone, suggesting that diffuse disease may have a distinct biology and clinical course compared to focal carcinosarcoma of the bone. Gemcitabine and docetaxel may be considered for similar patients.

## Data Availability

Not applicable.

## Abbreviations

SCC: squamous cell carcinoma
PET/CT: positron emission photography/computed tomography
EMT: epithelial to mesenchymal transition
MET: mesenchymal to epithelial transition
